# Association of Serum β-Hydroxybutyrate and Coronary Artery Disease in an Urban Chinese Population

**DOI:** 10.3389/fnut.2022.828824

**Published:** 2022-02-17

**Authors:** Hongna Mu, Ruiyue Yang, Siming Wang, Wenduo Zhang, Xinyue Wang, Hongxia Li, Jun Dong, Wenxiang Chen, Xue Yu, Fusui Ji

**Affiliations:** ^1^The Key Laboratory of Geriatrics, Beijing Institute of Geriatrics, Institute of Geriatric Medicine, Chinese Academy of Medical Sciences, Beijing Hospital/National Center of Gerontology of National Health Commission, Beijing, China; ^2^Department of Cardiology, Beijing Hospital, National Center of Gerontology, Institute of Geriatric Medicine, Chinese Academy of Medical Sciences, Beijing, China; ^3^National Center for Clinical Laboratories, Institute of Geriatric Medicine, Chinese Academy of Medical Sciences, Beijing Hospital/National Center of Gerontology, Beijing, China

**Keywords:** coronary artery disease, ketone bodies, β-hydroxybutyrate, Gensini score, metabolic dysfunction

## Abstract

Ketone bodies, including β-hydroxybutyrate (BHB), acetoacetate (AA), and acetone, can substitute and alternate with glucose under conditions of fuel/food deficiency. Ketone-body metabolism is increased in a myriad of tissue-metabolism disorders. Perturbations in metabolism are major contributors to coronary artery disease (CAD). We investigated the association of BHB with CAD. A total of 2,970 people of Chinese Han ethnicity were enrolled. The Gensini score was calculated for all patients who had positive findings. The serum level of BHB and other laboratory parameters were measured. The association of serum levels of metabolites with traditionally risk factors and CAD severity was analyzed. The BHB was found to be associated with some traditional risk factors of CAD and CAD severity, as determined by the Gensini score or the number of diseased regions. Moreover, BHB was associated with the T3/T1 tertiles of the Gensini score after the adjustment for traditional risk factors by multivariable logistic regression analysis. The association of BHB with CAD severity was more obvious in women. Taken together, these data suggest that the circulating BHB level is independently associated with CAD severity, and that this association is more pronounced in women.

## Introduction

Coronary artery disease (CAD) caused by atherosclerosis, has been reported to be the leading cause of death worldwide. Although new medical therapies have emerged in recent years, the risk of death from CAD is still high (1). Therefore, there is a need for the development of more accurate diagnostic methods, identification of novel biomarkers, more efficacious drugs and identification of new therapeutic targets to reduce the risk of death from CAD (2, 3).

Metabolic dysfunction is a hallmark of CAD pathophysiology, reflecting not only the altered metabolism of the myocardium but also the overall contributions from peripheral tissues and organs (4, 5). These metabolic changes influence disease pathophysiology directly, and may serve as earlier and more sensitive markers of CAD (6, 7). Abnormal accumulation or deficiency of specific metabolites within the circulation can be powerful markers in the management of CAD, with regard to the detecting disease, evaluating disease progression, and assessing therapeutic efficacy (8, 9).

Ketone bodies [e.g., β-hydroxybutyrate (BHB), acetoacetate (AA), and acetone] are water-soluble molecules that contain the ketone groups produced from fatty acids by the liver (ketogenesis). Ketone bodies are derived from increased beta oxidation of free fatty acid, BHB is a major component of the ketone body (10). During perturbed tissue metabolism, an increase in BHB may help circumvent the metabolic malfunctions that directly contribute to disease pathophysiology (11). Recent studies have shown that BHB level was increased in a myriad of pathological conditions, such as type 2 diabetes mellitus (T2DM) (12), arrhythmogenic cardiomyopathy (13), and heart failure (14). In addition, it was reported that BHB was increased in patients with non-ST segment elevation acute coronary syndrome, which reflects the oxidative stress and hypoxic state that myocardial cells suffer. Hence, BHB could be used for the early diagnosis of acute coronary syndrome (15). Obokata and colleagues showed that an increased serum level of BHB was independently associated with cardiovascular events and all-cause death in patients undergoing hemodialysis (16). Those studies implied a potential association of BHB with CAD. However, studies on the associations between BHB and CAD in Chinese Han populations have not been carried out. Here, we evaluated the association of BHB with CAD, as well as the severity of CAD, and risk factors of CAD in a case–control study.

## Materials and Methods

### Ethical Approval of the Study Protocol

The protocol for this cross-sectional study was approved (2016BJYYEC-121-03) by the Ethics Committee of Beijing Hospital (Beijing, China). Written informed consent was provided by all participants.

### Exclusion Criteria

The exclusion criteria were patients: (i) with severe congenital heart disease, severe cardiac insufficiency, primary pulmonary hypertension, hepatic/renal dysfunction, severe peripheral arterial disease, or related conditions which are contraindications to cardiac catheterization; (ii) receiving radiotherapy or chemotherapy; (iii) who were pregnant or nursing a baby; (iv) suffering from alcoholism or drug abuse; and (v) under treatment for mental illness.

### Study Cohort

Patients suspected of having CAD (or adjudicated to have a CAD history) and subjected to coronary angiography in Beijing Hospital between March 2017 and October 2020 were enrolled. The demographic characteristics (e.g., age and gender), medical history (e.g., DM, hypertension, and dyslipidemia), cigarette smoking, and body mass index (BMI) of the study cohort were recorded. Before coronary angiography, samples of venous blood were collected after an overnight fast. The serum was isolated, aliquoted, and stored at −80°C until analyses.

### Coronary Angiography

Coronary angiography was performed for CAD assessment by experienced cardiologists. All targeted coronary lesions of enrolled patients were analyzed by the built-in QCA software of the Allura Xper FD20 Angiography System (Philips Healthcare, Amsterdam, the Netherlands). Fifteen coronary segments were analyzed quantitatively based on the American Heart Association classification. According to current guidelines, “coronary stenosis” was defined as a reduction of >50% in the arterial diameter, and if this was not present, was diagnosed as non-CAD. In addition, individuals with negative findings on CT of the coronary arteries or stress myocardial perfusion imaging were categorized as the non-CAD group.

### Gensini Score

The Gensini score was used to assess the severity of damage to coronary arteries (17). The Gensini score was computed by assigning a severity score for each coronary stenosis depending on the degree of luminal narrowing and its importance based on location. Luminal stenoses of 25, 50, 75, 90, 99%, and total occlusion were given a Gensini score of 1, 2, 4, 8, 16, and 32, respectively. Then, these scores were multiplied by a factor according to location: 5, left main coronary artery; 2.5, proximal segment of the left anterior descending coronary artery and proximal segment of the circumflex artery; 1.5, mid-segment of the left anterior descending coronary artery; 1.0, right coronary artery, distal segment of the left anterior descending coronary artery, posterior descending artery, and obtuse marginal artery; and 0.5, other segments. Finally, the Gensini score was calculated by summation of the scores for individual coronary segments. The Gensini score was calculated in 2,047 patients who had not undergone percutaneous arterial intervention previously.

### Liquid Chromatography-Tandem Mass Spectrometry Measurement (LC-MS/MS) of the BHB Level

The serum concentration of BHB was quantified using an LC-MS/MS system comprising a 1,260 Infinity II Series high-performance liquid chromatography (HPLC) (Agilent, Santa Clara, CA, USA) coupled with a Sciex 5,500 QTRAP mass spectrometer (AB Sciex, Foster City, CA, USA). MS parameters were set as previously described (18). BHB standards, serum samples, and quality controls were, respectively, mixed with the isotopically labeled internal standard (BHB-D4) solution and proteins were precipitated with methanol containing 0.5% formic acid. After vortexing and centrifuging, the metabolites were separated by a Kinetex™ hydrophilic interaction LC column (2.6 μm, 150 mm × 2.1 mm, Phenomenex, Torrance, CA, USA) and eluted with a mobile phase of 75% methanol, containing 5 mmol/L ammonium formate, and 0.05% formic acid at a flow rate of 0.3 ml/min. BHB and internal standard were detected with positive electrospary ionization in multiple reaction monitor mode using characteristic precursor–product ion transitions of m/z 103.0 → 59.0 and m/z 107.0 → 59.0, respectively. The concentration of BHB was calculated using standard curves. The analytical coefficients of variation for BHB measurements were below 3%. Applied Biosystems Analyst version 1.6.2 software was used for system control, data collection, and processing.

### Other Parameters and Laboratory Testing

In addition, the serum samples were tested for fasting blood glucose (FBG), total cholesterol (TC), triglycerides (TG), high-density lipoprotein cholesterol (HDL-C), low-density lipoprotein cholesterol (LDL-C), creatinine (Crea), and uric acid (UA) using the respective assay kits (Sekisui Medical Technologies, Osaka, Japan) on a chemistry analyzer (7,180 series; Hitachi, Tokyo, Japan). Two quality control materials, prepared by mixed fresh serum samples, were analyzed with patient samples in each run in LC-MS/MS and the laboratory assays to monitor the performance of the measurements.

### Statistical Analysis

The normal distribution of samples was tested. Parameters with normal distribution are expressed as mean ± SD, while parameters with a skewed distribution are presented as the median and percentile (25–75th). Count data are reported as frequencies and percentages. The one-way ANOVA was conducted to compute the differences for continuous variables, while the non-parametric Jonckheere-Terpstra test was used to determine the differences when the data did not have a normal distribution. For categorical variables, the Chi-square test was used. Spearman's correlation analysis was employed to examine the associations between BHB and traditional CAD risk factors. The multivariable logistic regression analysis was employed to determine the relationship between the BHB level and the Gensini score. Potential confounding variables (age, gender, smoking status, obesity or overweight, hypertension, dyslipidemia, DM, history of stroke, and family history of premature CAD) were controlled in the regression models. Results are presented as odds ratios (ORs) and 95% *CI*s. The value of *p* < 0.05 (two-tailed) was considered significant. Statistical analysis was performed using SPSS 25.0 (IBM, Armonk, NY, USA).

## Results

### Clinical Characteristics

The study population comprised 2,970 patients (62.5% men). Patient characteristics at baseline according to the tertile of the BHB levels are summarized in Table 1. Individuals with a higher BHB level also had a higher systolic blood pressure (SBP), higher risk of a family history of premature CAD as well as a higher prevalence of DM and dyslipidemia. Furthermore, with the increase of BHB levels, FBG, TC, and LDL-C significantly increased (*p* < 0.001 for trend). People with a higher BHB level tended to be older (but not significantly so). Whereas, levels of BMI, diastolic blood pressure (DBP), TG, HDL-C, Crea, and UA were similar among the groups. In addition, associations were not found between the BHB level and overweight/obesity, hypertension, history of stroke, smoking status, or statins use.

**Table 1:** Comparison of baseline characteristics of study population according to serum BHB tertile.

**Characteristic^a^**	**Tertile of serum level of BHB (μM)**			
	**Low**	**Intermediate**	**High**	**Trend *p*-value**
*N*	989	992	989	–
Age, years	65.0 ± 10.6	65.7 ± 10.8	65.9 ± 11.3	0.058
Male, *n* (%)	638 (64.5)	618 (62.3)	599 (60.6)	0.066
BMI, kg/m2	25.7 ± 3.6	25.9 ± 3.3	25.5 ± 3.6	0.097
SBP, mmHg	135.6 ± 18.0	136.9 ± 18.5	137.6 ± 18.9	0.017
DBP, mmHg	78.6 ± 11.0	78.0 ± 11.1	78.4 ± 11.6	0.675
Overweight/obesity, *n* (%)	671 (67.8)	711 (71.7)	636 (64.3)	0.738
Hypertension, *n* (%)	659 (66.6)	673 (67.8)	668 (67.5)	0.809
Diabetes, *n* (%)	213 (21.5)	430 (43.3)	403 (40.7)	<0.001
Dyslipidemia, *n* (%)	398 (40.2)	474 (47.8)	440 (44.5)	0.041
History of stroke, *n* (%)	101 (10.2)	121 (12.2)	97 (9.8)	0.665
Family history of premature CAD, *n* (%)	87 (8.8)	69 (7.0)	73 (7.4)	0.041
**Smoking status**, ***n*** **(%)**				
Never	503 (50.9)	522 (52.6)	532 (53.8)	0.107
Former	152 (15.4)	145 (14.6)	157 (15.9)	
Current	330 (33.4)	316 (31.9)	298 (30.1)	
**Statins use**, ***n*** **(%)**				
No	551 (55.7)	525 (52.9)	575 (58.1)	0.727
Take statins intermittently	108 (10.9)	71 (7.2)	88 (8.9)	
Take statins continuously • Over 1 year, *n* (%)	238 (24.1)	293 (29.5)	223 (22.5)	
FBG, mmol/L	6.4 ± 2.1	7.0 ± 2.3	6.8 ± 2.3	<0.001
TC, mmol/L	3.8 ± 0.9	3.9 ± 0.9	4.0 ± 1.0	<0.001
TG, mmol/L	1.2 (0.9–1.7)	1.4 (1.0–1.9)	1.2 (0.9–1.7)	0.215
HDL-C, mmol/L	1.0 (0.9–1.2)	1.0 (0.9–1.2)	1.0 (0.9–1.2)	0.866
LDL-C, mmol/L	2.2 (1.7–2.7)	2.2 (1.7–2.8)	2.3 (1.8–2.9)	<0.001
Crea, μmol/L	72.0 ± 16.4	71.4 ± 16.9	71.6 ± 18.2	0.610
UA, μmol/L	323.0 ± 84.9	328.9 ± 85.7	329.0 ± 93.2	0.136
BHB, μmol/L	27.9 (21.1–34.6)	55.7 (48.0–66.3)	162.3 (110.5–315.4)	<0.001

*Not all data were available for all patients. BHB, β-hydroxybutyrate; CAD, coronary artery disease; BMI, body mass index; SBP, systolic blood pressure; DBP, diastolic blood pressure; FBG, fasting blood glucose; TC, total cholesterol; TG, triglyceride; HDL-C, high-density lipoprotein cholesterol; LDL-C, low-density lipoprotein cholesterol; Crea, creatinine; UA, uric acid*.

a *Data are mean ± SD, median (interquartile range) for continuous variables, or percentage for categorical variables*.

### Correlations of Serum BHB Level With the Traditional CAD Risk Factors

A significant positive correlation was found between the serum BHB level and the level of FBG (*r* = 0.067, *p* < 0.001), TC (*r* = 0.070, *p* < 0.001), and LDL-C (*r* = 0.066, *p* = 0.001). The serum BHB level was significantly positively correlated with age (*r* = 0.041, *p* < 0.05) and SBP (*r* = 0.040, *p* < 0.05). However, an association between the BHB level and level of DBP, TG, HDL-C, Crea, UA, and BMI was not significant (Table 2).

**Table 2:** Spearman's correlation of BHB with traditional CAD risk factors.

	**Correlation coefficients**	***P***
Age	0.041	0.027
BMI	−0.032	0.087
SBP	0.040	0.028
DBP	−0.011	0.542
FBG	0.067	<0.001
TC	0.070	<0.001
TG	0.019	0.338
HDL-C	0.010	0.596
LDL-C	0.066	0.001
Crea	−0.017	0.377
UA	0.021	0.260

*CAD, coronary artery disease; BMI, body mass index; SBP, systolic blood pressure; DBP, diastolic blood pressure; FBG, fasting blood glucose; TC, total cholesterol; TG, triglyceride; HDL-C, high-density lipoprotein cholesterol; LDL-C, low-density lipoprotein cholesterol; Crea, creatinine; UA, uric acid*.

### Relationship Between the BHB Level and CAD Severity

**Figure 1:**
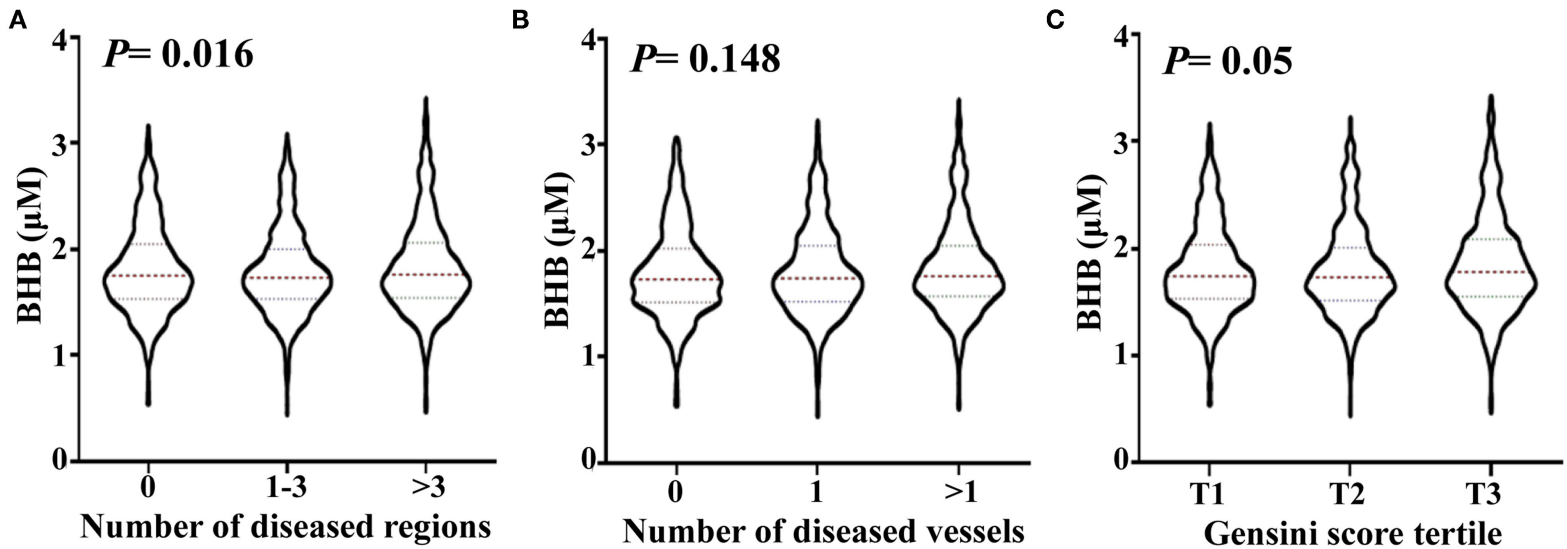
Association of the serum BHB level with the severity of coronary artery lesion. Violin plots of serum BHB concentrations at presentation with the number of stenosed regions **(A)**, the number of stenosed vessels **(B)** and tertile of the Gensini scores **(C)**, showing median (red dashed line) and interquartile ranges (blue dashed line) on a log 10 scale. *p* < 0.05 considered statistically significant.

To analyze the relationship between the BHB level and CAD severity, patients with positive findings upon coronary angiography were classified into subgroups for those with 1–3 and >3 stenosed regions. The BHB level was significantly increased (*p*_trend_ = 0.016) with an increase in the number of stenosed regions. Then, the patients with CAD were divided into subgroups with 1 stenosed or >1 stenosed vessels. As shown in Figure 1, no significant change was found for the BHB level among groups. The Gensini score is widely used to evaluate the severity of coronary atherosclerosis, hence the studied patients were divided into different subgroups according to tertile of the Gensini score: the BHB level was associated with the Gensini score (*p*_trend_ = 0.05).

### The Multivariable Logistic Regression Analysis of the BHB Level With CAD

Multivariate logistic regression analysis was done to examine the independent relationship between the BHB level and CAD severity. Here, we adopted tertiles of T3/T1 of the Gensini score to represent CAD severity. As shown in Table 3, the crude *OR* was increased with increasing BHB levels in Model 1 (*p* = 0.004). After adjustment for age and gender (Model 2), high BHB levels were significantly associated with CAD severity, with *OR* (95% *CI*) of 1.178 (1.045–1.327), *p* = 0.007. In Model 3, traditional risk factors of CAD [age, smoking (former and current smoking), hypertension, DM, and family history of premature CAD] were all positively associated with the Gensini score, whereas being female was a protective factor for CAD, with *OR* of 0.417 (0.308–0.564). An increased level of BHB continued to be independently associated with T3/T1 tertiles of the Gensini score (*OR* = 1.151, 95% *CI*: 1.012–1.309, *p* = 0.032), after adjustment for traditional risk factors, such as age, gender, smoking status, overweight/obesity, hypertension, dyslipidemia, DM, history of stroke, and family history of premature CAD (Table 3).

**Table 3:** Odds ratios (95% *CI*s) and *p* for the severity of coronary artery lesion.

		***P*-value**	**OR (95% confidence intervals)**
Model 1^a^	BHB	0.004	1.177 (1.053–1.314)
Model 21^b^	Age	<0.001	1.037 (1.026–1.048)
	Gender	<0.001	0.304 (0.240–0.386)
	BHB	0.007	1.178 (1.045–1.327)
Model 31^c^	Age	<0.001	1.035 (1.023–1.048)
	Gender	<0.001	0.417 (0.308–0.564)
	**Smoking status**		
	Never	–	–
	Former	0.016	1.689 (1.103–2.587)
	Current	<0.001	2.082 (1.503–2.883)
	Overweight/obesity	0.102	0.802 (0.616–1.045)
	Hypertension	0.020	1.353 (1.048–1.746)
	Dyslipidemia	0.877	0.981 (0.774–1.245)
	Diabetes	<0.001	2.570 (1.979–3.339)
	History of stroke	0.241	1.266 (0.853–1.878)
	Family history of premature CAD	0.011	1.504 (1.097–2.063)
	BHB	0.032	1.151 (1.012–1.309)

a *Model 1: Crude risk*.

b *Model 2: Adjusted for age and gender*.

c *Model 3: Further adjusted for smoking status, obesity or overweight, hypertension, dyslipidemia, diabetes, stroke, and family history of premature CAD. For gender, smoking status, overweight/obesity, hypertension, dyslipidemia, diabetes, history of stroke, family history of premature CAD, the reference are women, never smoking, body mass index (BMI) <24 kg/m^2^, no hypertension, no dyslipidemia, no diabetes, no history of stroke, and no family history of premature CAD, respectively*.

### The Stratified Analysis of the BHB Level With CAD

**Figure 2:**
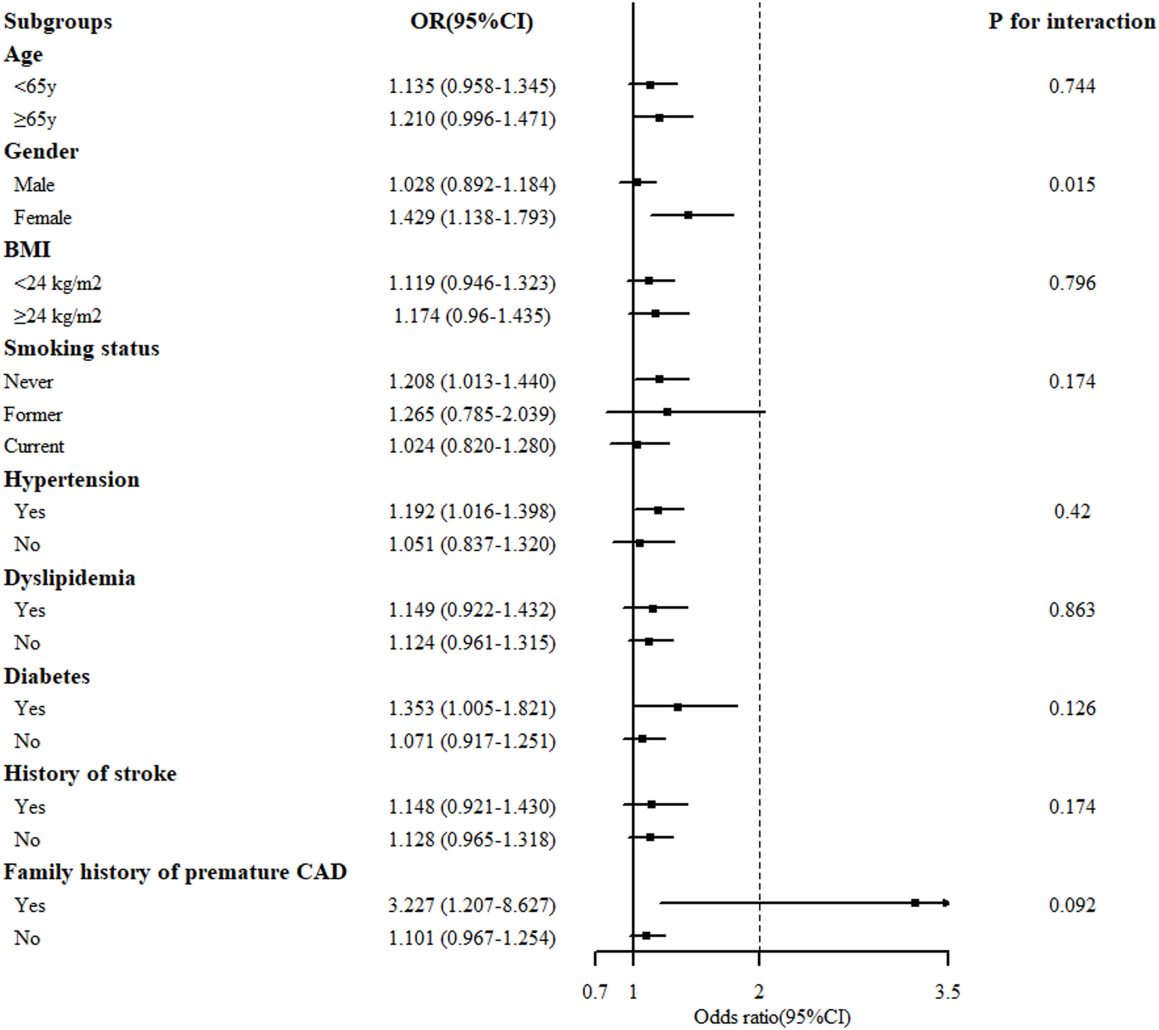
Stratified analysis of the association [odds ratio (*OR*) (95% *CI*)] between the circulating BHB level (per 1-SD increment) and the severity of coronary artery lesion. Values are adjusted for age, gender, smoking status, obesity or overweight, hypertension, dyslipidemia, diabetes, stroke, and family history of premature CAD, stratifying factors excepted. The *p* < 0.05 considered statistically significant.

**Figure 3:**
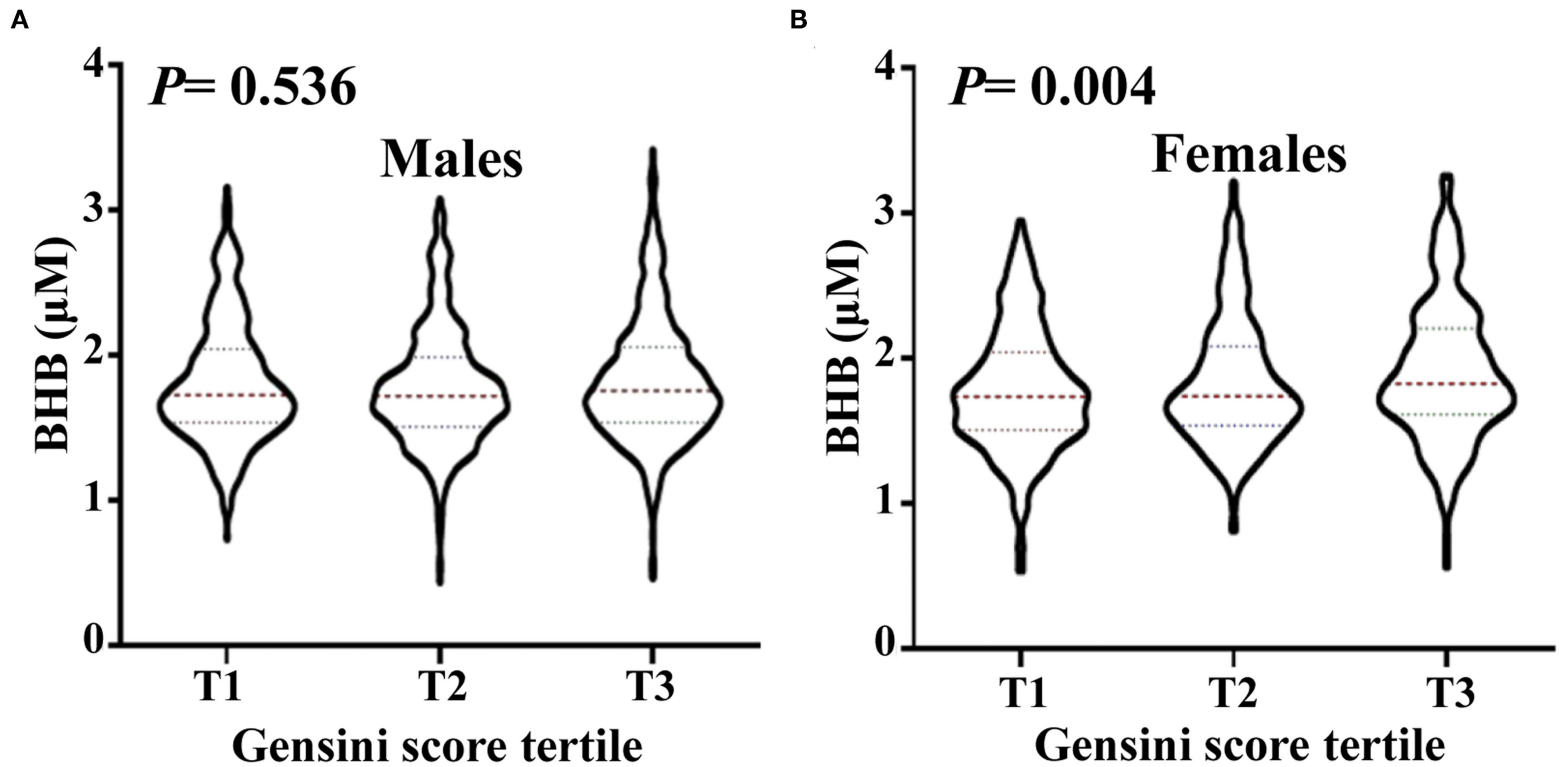
The level of circulating BHB in men and women, respectively, according to Gensini score tertile. Violin plots of serum BHB concentrations in men **(A)** and women **(B)** by Gensini score tertile, showing median (red dashed line) and interquartile ranges (blue dashed line) on a log10 scale. The *p* < 0.05 considered statistically significant.

Stratified analysis of the association of the serum BHB level (per 1-SD increment) and the severity of coronary artery lesion was conducted in different groups of age, gender, BMI, smoking status, hypertension, dyslipidemia, DM, history of stroke, and family history of premature CAD. The interaction between the BHB level and these factors on CAD distribution was analyzed. As presented in Figure 2, there was a significant association between the BHB level and CAD in women, non-smokers, people with hypertension, DM, or a family history of premature CAD. Moreover, we observed that the association between the BHB level and CAD was modified by gender (*P*_interaction_ = 0.015). The BHB level was associated with CAD in women (*OR*: 1.429, 95% *CI*: 1.138–1.793, *p* = 0.002), but not in men (*OR*: 1.028, 95% *CI*: 0.892–1.1840, *p* = 0.704), after adjustment for age, BMI, smoking status, hypertension, dyslipidemia, DM, history of stroke, and family history of premature CAD. However, an interaction was not observed between the BHB level and age, BMI, smoking status, hypertension, dyslipidemia, DM, history of stroke or family history of premature CAD (Figure 2). Then, we further analyzed the association of the BHB level with CAD severity in men and women, respectively, according to the Gensini score. Consistent with this, the BHB level showed a significant association with the Gensini score in women, but not in men (Figure 3).

## Discussion

**Figure 4:**
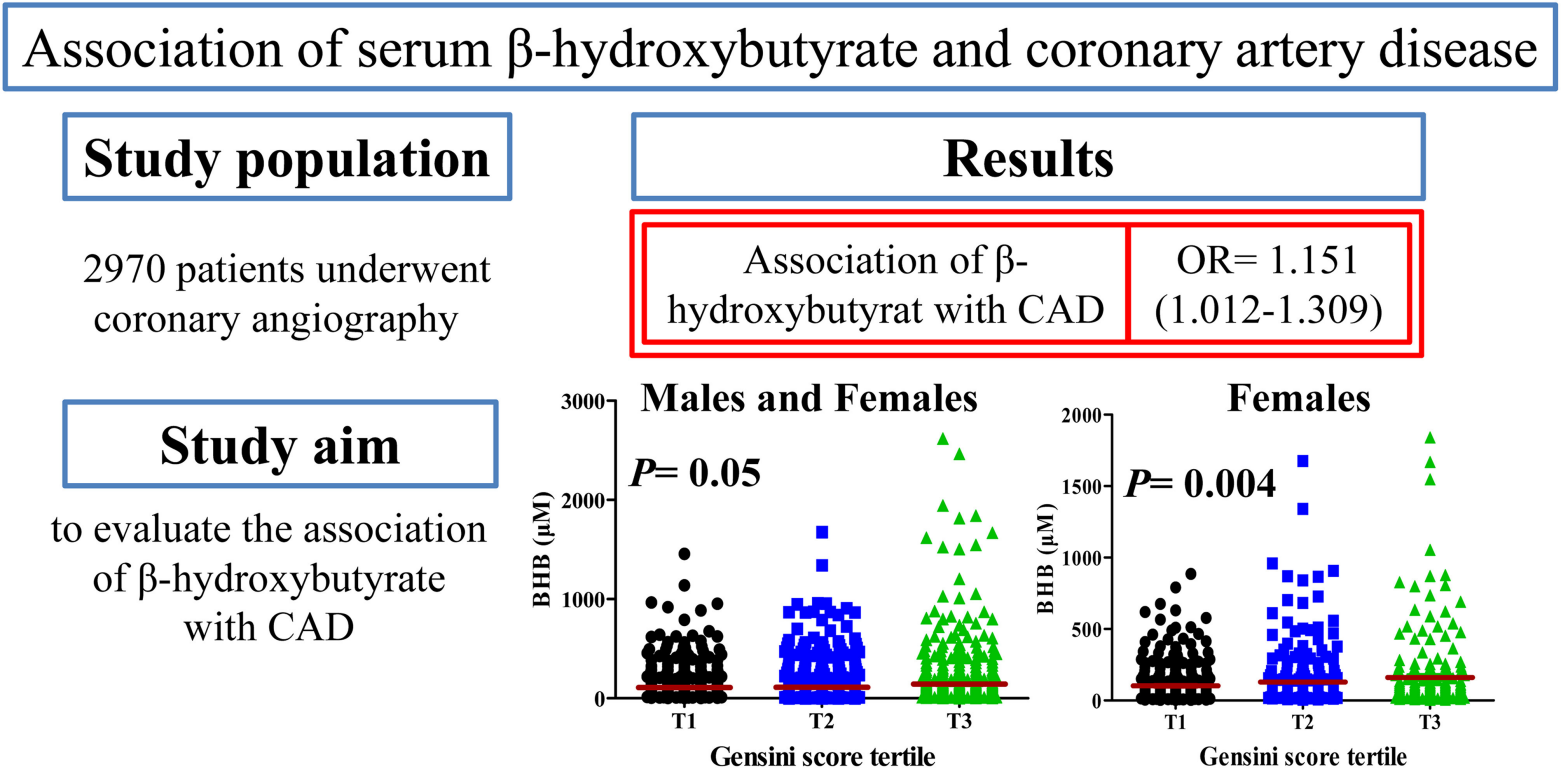
Overview of this study. CAD, coronary artery disease; OR, odds ratio.

In this study, we observed circulating BHB concentration was associated with the traditional risk factors and severity of CAD. An increased BHB level was associated with increased CAD severity, as defined by Gensini score or number of stenosed regions. This association of the BHB level with the Gensini score remained significant after adjustment for the conventional risk factors of CAD by multivariable logistic regression analysis. An increased level of BHB in patients with CAD indicated a more severe state of the disease and more severe systemic metabolic perturbations, especially in women (Figure 4). To the best of our knowledge, this study is the first to report that an increased BHB level to be associated with an increased risk of CAD in a Chinese population.

The heart is the most metabolically demanding organ in the body, so impaired metabolism may lead to CAD, thus, the identification and quantification of changes in levels of these metabolites is critical for the diagnosis and treatment of CAD (19). Ketone bodies, such as BHB, AA, and acetone, are “small fuel substrates,” derived from increased beta oxidation of free fatty acid. These substances can uniquely substitute and alternate with glucose under conditions of fuel/food deficiency (20). There is considerable evidence that the level of ketone bodies, in particular BHB, is increased in metabolic disorders. The most robust evidence is from a retrospective cohort study which demonstrates that the BHB level is independently and significantly associated with adverse cardiovascular events and therefore could serve as a novel biomarker predicting the survival and risk of cardiovascular events in patients undergoing hemodialysis (16). Furthermore, the circulating level of ketone bodies is increased in patients presenting with ST-segment elevation myocardial infarction and a higher level of ketone bodies after 24 h is associated with functional outcomes. Hence, the increased metabolism of ketones may play a part in the response to myocardial ischemia (21). Accumulating evidence has shown that the BHB level is increased in patients with heart failure (14, 22), obese and/or T2DM (12, 23). Of importance, with metabolic dysfunction as an inherent feature of the CAD pathophysiology, whether circulating BHB is increased in CAD remains poorly understood. We investigated if the BHB level is associated with CAD in a Chinese Han population.

First we analyzed the association between the circulating BHB concentrations with CAD traditional risk factors. The result revealed a strong association between the BHB level with the concentration of FBG, TC, and LDL-C, as well as a significant association between the BHB concentration and age and SBP. These results were not in line with some of the previous reports (13, 24), possibly on account of different populations studied. High TC and high LDL-C all play a role in CAD (25–28). We discovered a significant association between BHB with TC and LDL-C (Table 2). However, multivariable logistic regression analysis revealed dyslipidemia not to be associated with CAD severity. On account of the use of statins therapy, the relationship between the clinical diagnosis of dyslipidemia and CAD was not significant.

Next, we analyzed the relation of BHB with CAD and CAD severity. The study population was first divided into the non-CAD group and CAD group according to coronary angiography. People who developed CAD did not have a significantly higher BHB level in serum compared with that in healthy controls (data not shown). In a pilot study undertaken in Japan, Omori et al. found the BHB level to be significantly lower in the CAD group compared with that in the control subjects (in patients with DM), but the study cohorts were quite small (5). To analyze the association between the BHB level and CAD severity, the CAD group was divided into subgroups, based on the number of stenosed vessels or regions: the BHB level was associated with the severity of CAD according to the number of stenosed regions. Gensini score is widely used for evaluating the coronary atherosclerotic severity with the judgement of the position, number, and stenosis of blood vessels caused by coronary atherosclerosis (17). The CAD patients were divided into 3 groups according to the Gensini score tertile, and the BHB level showed a significant association with the Gensini score tertile (Figure 1). Multivariable logistic regression analysis revealed the BHB level to be independently associated with the T3/T1 tertiles of the Gensini score, even after adjustment for other well-known risk factors (Table 3).

Additional stratification analysis revealed the BHB level to have a strong association with CAD in women (Figure 2). Furthermore, in accordance with this, Figure 3 revealed that the BHB level was associated with CAD severity in women not men, according to the Gensini score. These results indicated that BHB may be involved in the progress of atherosclerosis especially in women. Actually, cardiac metabolism and the burden of risk factors differ by gender (29, 30), gender may have an influence on both the development and progression of CAD and on the pattern of compositional plaque progression, and women have a higher prevalence of mortality from CAD than men (31). Thus, the role of BHB in the development and progression of CAD in women merits further investigation.

Traditionally, severe ketosis, or ketoacidosis, is known as a harmful event. Ketone bodies are synthesized in the liver from acetyl-coenzyme-A derived primarily from fatty acid β-oxidation and are transported to extrahepatic tissues for terminal oxidation as a source for adenosine triphosphate generation at the expense of glucose oxidation (32). Myocardial ischemia occurs when coronary blood flow is inadequate, and hence, the oxygen supply to the myocardium is not sufficient to meet the oxygen demand. Ischemia elicits disturbances in the balance between energy metabolism and cardiac function (33). It seems that hepatic ketogenesis is activated and cardiac utilization is not enhanced, the concentration of blood ketone bodies is highly increased in patients with ischemic heart disease (29, 33). In addition, people suffering from DM not only have increased production of ketones, their ketone clearance is also decreased significantly, because of low insulin levels, and decreased activities of succinyl-coenzyme-A: 3 oxoacid coenzyme-A-transferase and β-hydroxybutyrate dehydrogenase, which all contribute to an increased level of ketones (34). DM is a major risk factor for CAD, and many patients with DM have CAD, which is frequently associated with insulin resistance; a similar scenario has been noted for patients with CAD with an increased level of BHB (35, 36). A clinical study also supports this concept: patients with CAD have deficits in metabolic fuel extraction, with reduced extraction of fatty acid and ketone compared with that in controls (37). What's more, an increased level of BHB has an effect on upregulating expression of intercellular adhesion molecule-1 in endothelial cells (38), increasing the secretion of tumor necrosis factor-alpha in cultured U937 monocytes (39), and promoting lipid peroxidation levels in patients with DM (40). These phenomena would be implicated in the development of vascular disease and atherosclerosis (34).

However, recent evidence from experimental studies and clinical research has uncovered a protective role for ketones in cardiovascular disease. BHB infusion has been shown to specifically improve working memory performance in patients with T2DM in the absence of changes in global cognition (41). Even in healthy people, exogenous administration of ketones has been shown to improve the cardiac function (42). In addition, data from animal experiments have shown that ketones aid the prevention and treatment of heart failure by reducing inflammation (43), normalizing myocardial adenosine triphosphate production (44), and ameliorating pathologic cardiac remodeling and dysfunction (45). A hepatocyte-macrophage “AA shuttle” has been shown to coordinate the fibrogenic response to hepatic injury *via* mitochondrial metabolism in tissue macrophages after high fat diet-induced fibrosis (46). Furthermore, BHB has been found to protect the heart from ischemia/reperfusion injury (47) and attenuate atherosclerosis (48) in different mice models. Indeed, ketone bodies possess various capabilities and participate in multiple cellular processes (49), especially in cardiovascular disease, further studies to ascertain the action and specific mechanism of BHB in CAD are warranted.

During fast, DM and other pathological conditions, ketone bodies are all increased, the blood levels of BHB can be used to reflect ketotic state of a patient and American Diabetes Association prefers quantitative determination of blood BHB for the diagnosis and follow-up of ketoacidosis (50, 51). In ketone bodies, BHB is the most abundant in mammals. BHB has been found at a higher concentration than that of AA and acetone in subjects of DM and myocardial infarction. In cohort studies, BHB is the ketone body studied most often (21, 34, 52). Therefore, we focused only on BHB in this study, which is one limitation. The serum level of acetone is very low and the potential role of acetone in CAD is poorly known. AA is the central ketone body, and the other ketone bodies are derived from it. An increased level of AA has a role in oxidative modification of LDLs and then causes impaired cholesterol uptake and its deposition in the arterial wall and atherosclerosis in patients with DM (53). AA can also increase interleukin-6 secretion in U937 monocytes (54), upregulate expression of intercellular adhesion molecule-1 (55), facilitate the activity of NADPH oxidase (56), and promote glutathione depletion (57) in human umbilical vein endothelial cells. Those actions aggravate the inflammatory response, monocyte adhesion, and oxidative stress and contribute to the initiation and progression of CAD.

In this study, the control group already had high levels of cardiovascular risk factors and these people did not represent a true healthy control, which may have led to underestimation of the association between the metabolites and CAD and limited the power of this study. This is another limitation of the study.

Third, we did not adequately address that BHB as a biomarker for CAD can be an adaptive response that lack of enough evidence. Additionally, this study was a cross-sectional study, and we only addressed the association of BHB with CAD, this association does not inform causality. Therefore, further studies are necessary and the findings need to be confirmed in future prospective analysis. This is also a limitation of our study.

## Conclusion

The circulating level of BHB was independently associated with CAD severity, and a gender-related difference in this association was documented. Further studies are needed to determine the exact role of BHB in CAD.

## Data Availability Statement

The original contributions presented in the study are included in the article/supplementary material, further inquiries can be directed to the corresponding author/s.

## Ethics Statement

The studies involving human participants were reviewed and approved by the Ethics Committee of Beijing Hospital. The patients/participants provided their written informed consent to participate in this study.

## Author Contributions

HM, FJ, and XY designed the research and drafted the article. HM, RY, and SW measured BHB in serum, performed the biochemical assays, and processed the data. WZ and XW performed coronary angiography and calculated the Gensini score. HL, JD, and WC calculated the number of stenosed vessels and regions. FJ and XY revised the manuscript. All authors contributed to the article and approved the submitted version.

## Funding

This work was supported by the Beijing Natural Science Foundation (7214250), the National Key R&D Program of China (2020YFC2008304), the Non-profit Central Research Institute Fund of the Chinese Academy of Medical Sciences (2019TX310001), and the Beijing Hospital Special Fund Project (BJ-2019-184).

## Conflict of Interest

The authors declare that the research was conducted in the absence of any commercial or financial relationships that could be construed as a potential conflict of interest.

## Publisher's Note

All claims expressed in this article are solely those of the authors and do not necessarily represent those of their affiliated organizations, or those of the publisher, the editors and the reviewers. Any product that may be evaluated in this article, or claim that may be made by its manufacturer, is not guaranteed or endorsed by the publisher.

## Abbreviations

BHB: β-hydroxybutyrate
BMI: body mass index
CAD: coronary artery disease
Crea: creatinine
DBP: diastolic blood pressure
DM: diabetes mellitus
FBG: fasting blood glucose
HDL-C: high density lipoprotein cholesterol
LDL-C: low density lipoprotein cholesterol
SBP: systolic blood pressure
TC: total cholesterol
TG: triglyceride
UA: uric acid.

## References

[1] Senthong V Li XS Hudec T Coughlin J Wu Y Levison B. Plasma trimethylamine N-Oxide, a gut microbe-generated phosphatidylcholine metabolite, is associated with atherosclerotic burden. J Am Coll Cardiol. (2016) 67:2620–8. 10.1016/j.jacc.2016.03.546 27256833 PMC4893167

[2] Tunon J Barbas C Blanco-Colio L Burillo E Lorenzo O Martin-Ventura JL. Proteomics and metabolomics in biomarker discovery for cardiovascular diseases: progress and potential. Expert Rev Proteomics. (2016) 13:857–71. 10.1080/14789450.2016.1217775 27459711

[3] Kordalewska M Markuszewski MJ. Metabolomics in cardiovascular diseases. J Pharm Biomed Anal. (2015) 113:121–36. 10.1016/j.jpba.2015.04.021 25958299

[4] Franssens BT Van Der Graaf Y Kappelle LJ Westerink J De Borst GJ Cramer MJ. Body weight, metabolic dysfunction, and risk of type 2 diabetes in patients at high risk for cardiovascular events or with manifest cardiovascular disease: a cohort study. Diabetes Care. (2015) 38:1945–51. 10.2337/dc15-0684 26307608

[5] Omori K Katakami N Yamamoto Y Ninomiya H Takahara M Matsuoka TA. Identification of metabolites associated with onset of CAD in diabetic patients using CE-MS analysis: a pilot study. J Atheroscler Thromb. (2019) 26:233–45. 10.5551/jat.42945 30068816 PMC6402886

[6] Ussher JR Elmariah S Gerszten RE Dyck JR. The emerging role of metabolomics in the diagnosis and prognosis of cardiovascular disease. J Am Coll Cardiol. (2016) 68:2850–70. 10.1016/j.jacc.2016.09.972 28007146

[7] Wang Z Zhao Y. Gut microbiota derived metabolites in cardiovascular health and disease. Protein Cell. (2018) 9:416–31. 10.1007/s13238-018-0549-0 29725935 PMC5960473

[8] Dona AC Coffey S Figtree G. Translational and emerging clinical applications of metabolomics in cardiovascular disease diagnosis and treatment. Eur J Prev Cardiol. (2016) 23:1578–89. 10.1177/2047487316645469 27103630

[9] Chen ZZ Gerszten RE. Metabolomics and proteomics in type 2 diabetes. Circ Res. (2020) 126:1613–27. 10.1161/CIRCRESAHA.120.315898 32437301 PMC11118076

[10] Moller N. Ketone body, 3-hydroxybutyrate: minor metabolite - major medical manifestations. J Clin Endocrinol Metab. (2020) 105:dgaa370. 10.1210/clinem/dgaa370 32525972

[11] Schugar RC Moll AR Andre D'avignon D Weinheimer CJ Kovacs A Crawford PA. Cardiomyocyte-specific deficiency of ketone body metabolism promotes accelerated pathological remodeling. Mol Metab. (2014) 3:754–69. 10.1016/j.molmet.2014.07.010 25353003 PMC4209361

[12] Vigili De Kreutzenberg S Avogaro A. The role of point-of-care 3-hydroxybutyrate testing in patients with type 2 diabetes undergoing coronary angiography. J Endocrinol Invest. (2017) 40:627–34. 10.1007/s40618-017-0615-0 28188583 PMC5443872

[13] Song JP Chen L Chen X Ren J Zhang NN Tirasawasdichai T. Elevated plasma beta-hydroxybutyrate predicts adverse outcomes and disease progression in patients with arrhythmogenic cardiomyopathy. Sci Transl Med. (2020) 12:1–13. 10.1126/scitranslmed.aay8329 32051229

[14] Flores-Guerrero JL Westenbrink BD Connelly MA Otvos JD Groothof D Shalaurova I. Association of beta-hydroxybutyrate with development of heart failure: sex differences in a dutch population cohort. Eur J Clin Invest. (2021) 51:e13468. 10.1111/eci.13468 33616911 PMC8244065

[15] Laborde CM Mourino-Alvarez L Posada-Ayala M Alvarez-Llamas G Serranillos-Reus MG Moreu J. Plasma metabolomics reveals a potential panel of biomarkers for early diagnosis in acute coronary syndrome. Metabolomics. (2014) 10:414–24. 10.1007/s11306-013-0595-9 25814918 PMC4363481

[16] Obokata M Negishi K Sunaga H Ishida H Ito K Ogawa T. Association between circulating ketone bodies and worse outcomes in hemodialysis patients. J Am Heart Assoc. (2017) 6:1–9. 10.1161/JAHA.117.006885 28974498 PMC5721877

[17] Gensini GG. A more meaningful scoring system for determining the severity of coronary heart disease. Am J Cardiol. (1983) 51:606. 10.1016/s0002-9149(83)80105-2 6823874

[18] Tang Y Wang S Zhang W Yang R Yu X Wang X. A single-run, rapid polarity switching method for simultaneous quantification of cardiovascular disease-related metabolites using liquid chromatography–tandem mass spectrometry. Int J Mass Spec. (2021) 461:1–9. 10.1016/j.ijms.2020.116500

[19] Chen XF Chen X Tang X. Short-chain fatty acid, acylation and cardiovascular diseases. Clin Sci. (2020) 134:657–76. 10.1042/CS20200128 32219347

[20] Nielsen R Moller N Gormsen LC Tolbod LP Hansson NH Sorensen J. Cardiovascular effects of treatment with the ketone body 3-hydroxybutyrate in chronic heart failure patients. Circulation. (2019) 139:2129–41. 10.1161/CIRCULATIONAHA.118.036459 30884964 PMC6493702

[21] De Koning MLY Westenbrink BD Assa S Garcia E Connelly MA Van Veldhuisen DJ. Association of circulating ketone bodies with functional outcomes after ST-segment elevation myocardial infarction. J Am Coll Cardiol. (2021) 78:1421–32. 10.1016/j.jacc.2021.07.054 34593124

[22] Du Z Shen A Huang Y Su L Lai W Wang P. 1H-NMR-based metabolic analysis of human serum reveals novel markers of myocardial energy expenditure in heart failure patients. PLoS ONE. (2014) 9:e88102. 10.1371/journal.pone.0088102 24505394 PMC3914925

[23] Mahendran Y Vangipurapu J Cederberg H Stancakova A Pihlajamaki J Soininen P. Association of ketone body levels with hyperglycemia and type 2 diabetes in 9,398 Finnish men. Diabetes. (2013) 62:3618–26. 10.2337/db12-1363 23557707 PMC3781437

[24] Stryeck S Gastrager M Degoricija V Trbusic M Potocnjak I Radulovic B. Serum concentrations of citrate, tyrosine, 2- and 3- hydroxybutyrate are associated with increased 3-month mortality in acute heart failure patients. Sci Rep. (2019) 9:6743. 10.1038/s41598-019-42937-w 31043697 PMC6494857

[25] Miller M Seidler A Kwiterovich PO Pearson TA. Long-term predictors of subsequent cardiovascular events with coronary artery disease and 'desirable' levels of plasma total cholesterol. Circulation. (1992) 86:1165–70. 10.1161/01.cir.86.4.1165 1394924

[26] Mostaza JM Gomez MV Gallardo F Salazar ML Martin-Jadraque R Plaza-Celemin L. Cholesterol reduction improves myocardial perfusion abnormalities in patients with coronary artery disease and average cholesterol levels. J Am Coll Cardiol. (2000) 35:76–82. 10.1016/s0735-1097(99)00529-x 10636263

[27] Maher VM Brown BG Marcovina SM Hillger LA Zhao XQ Albers JJ. Effects of lowering elevated LDL cholesterol on the cardiovascular risk of lipoprotein(a). JAMA. (1995) 274:1771–4. 7500507

[28] O'donoghue ML Morrow DA Tsimikas S Sloan S Ren AF Hoffman EB. Lipoprotein(a) for risk assessment in patients with established coronary artery disease. J Am Coll Cardiol. (2014) 63:520–7. 10.1016/j.jacc.2013.09.042 24161323 PMC3945105

[29] Arima Y Izumiya Y Ishida T Takashio S Ishii M Sueta D. Myocardial ischemia suppresses ketone body utilization. J Am Coll Cardiol. (2019) 73:246–7. 10.1016/j.jacc.2018.10.040 30408507

[30] Hemal K Pagidipati NJ Coles A Dolor RJ Mark DB Pellikka PA. Sex differences in demographics, risk factors, presentation, and noninvasive testing in stable outpatients with suspected coronary artery disease: insights from the PROMISE trial. JACC Cardiovasc Imag. (2016) 9:337–46. 10.1016/j.jcmg.2016.02.001 27017234 PMC4982809

[31] Lee SE Sung JM Andreini D Al-Mallah MH Budoff MJ Cademartiri F. Sex differences in compositional plaque volume progression in patients with coronary artery disease. JACC Cardiovasc Imag. (2020) 13:2386–96. 10.1016/j.jcmg.2020.06.034 32828763

[32] Cotter DG Schugar RC Crawford PA. Ketone body metabolism and cardiovascular disease. Am J Physiol Heart Circ Physiol. (2013) 304:H1060–76. 10.1152/ajpheart.00646.2012 23396451 PMC3625904

[33] Lopaschuk GD Ussher JR Folmes CD Jaswal JS Stanley WC. Myocardial fatty acid metabolism in health and disease. Physiol Rev. (2010) 90:207–58. 10.1152/physrev.00015.2009 20086077

[34] Kanikarla-Marie P Jain SK. Hyperketonemia and ketosis increase the risk of complications in type 1 diabetes. Free Radic Biol Med. (2016) 95:268–77. 10.1016/j.freeradbiomed.2016.03.020 27036365 PMC4867238

[35] Naito R Miyauchi K. Coronary artery disease and type 2 diabetes mellitus. Int Heart J. (2017) 58:475–80. 10.1536/ihj.17-191 28717115

[36] Rutter MK Meigs JB Sullivan LM D'agostino RB Sr. Wilson PW. Insulin resistance, the metabolic syndrome, and incident cardiovascular events in the framingham offspring study. Diabetes. (2005) 54:3252–7. 10.2337/diabetes.54.11.3252 16249452

[37] Turer AT Stevens RD Bain JR Muehlbauer MJ Van Der Westhuizen J Mathew JP. Metabolomic profiling reveals distinct patterns of myocardial substrate use in humans with coronary artery disease or left ventricular dysfunction during surgical ischemia/reperfusion. Circulation. (2009) 119:1736–46. 10.1161/CIRCULATIONAHA.108.816116 19307475 PMC2756963

[38] Rains JL Jain SK. Hyperketonemia increases monocyte adhesion to endothelial cells and is mediated by LFA-1 expression in monocytes and ICAM-1 expression in endothelial cells. Am J Physiol Endocrinol Metab. (2011) 301:E298–306. 10.1152/ajpendo.00038.2011 21540444 PMC3154536

[39] Jain SK Kannan K Lim G Mcvie R Bocchini Jr JA. Hyperketonemia increases tumor necrosis factor-alpha secretion in cultured U937 monocytes and Type 1 diabetic patients and is apparently mediated by oxidative stress and cAMP deficiency. Diabetes. (2002) 51:2287–93. 10.2337/diabetes.51.7.2287 12086962

[40] Jain SK Mcvie R Jackson R Levine SN Lim G. Effect of hyperketonemia on plasma lipid peroxidation levels in diabetic patients. Diabetes Care. (1999) 22:1171–5. 10.2337/diacare.22.7.1171 10388984

[41] Jensen NJ Nilsson M Ingerslev JS Olsen DA Fenger M Svart M. Effects of beta-hydroxybutyrate on cognition in patients with type 2 diabetes. Eur J Endocrinol. (2020) 182:233–42. 10.1530/EJE-19-0710 31821157

[42] Selvaraj S Hu R Vidula MK Dugyala S Tierney A Ky B. Acute echocardiographic effects of exogenous ketone administration in healthy participants. J Am Soc Echocardiogr. (2021). 10.1016/j.echo.2021.10.017. [Epub ahead of print]. 34798244 PMC8901445

[43] Byrne NJ Soni S Takahara S Ferdaoussi M Al Batran R Darwesh AM. Chronically elevating circulating ketones can reduce cardiac inflammation and blunt the development of heart failure. Circ Heart Fail. (2020) 13:e006573. 10.1161/CIRCHEARTFAILURE.119.006573 32493060

[44] Yurista SR Matsuura TR Sillje HHW Nijholt KT Mcdaid KS Shewale SV. Ketone ester treatment improves cardiac function and reduces pathologic remodeling in preclinical models of heart failure. Circ Heart Fail. (2021) 14:e007684. 10.1161/CIRCHEARTFAILURE.120.007684 33356362 PMC7819534

[45] Horton JL Davidson MT Kurishima C Vega RB Powers JC Matsuura TR. The failing heart utilizes 3-hydroxybutyrate as a metabolic stress defense. JCI Insight. (2019)4. 10.1172/jci.insight.124079 30668551 PMC6478419

[46] Puchalska P Martin SE Huang X Lengfeld JE Daniel B Graham MJ. Hepatocyte-macrophage acetoacetate shuttle protects against tissue fibrosis. Cell Metab. (2019) 29:383–98 e387. 10.1016/j.cmet.2018.10.015 30449686 PMC6559243

[47] Yu Y Yu Y Zhang Y Zhang Z An W Zhao X. Treatment with D-beta-hydroxybutyrate protects heart from ischemia/reperfusion injury in mice. Eur J Pharmacol. (2018) 829:121–8. 10.1016/j.ejphar.2018.04.019 29679541

[48] Zhang SJ Li ZH Zhang YD Chen J Li Y Wu FQ. Ketone body 3-hydroxybutyrate ameliorates atherosclerosis via receptor Gpr109a-mediated calcium influx. Adv Sci. (2021) 8:2003410. 10.1002/advs.202003410 33977048 PMC8097358

[49] Yurista SR Chong CR Badimon JJ Kelly DP De Boer RA Westenbrink BD. Therapeutic potential of ketone bodies for patients with cardiovascular disease: JACC state-of-the-art review. J Am Coll Cardiol. (2021) 77:1660–9. 10.1016/j.jacc.2020.12.065 33637354

[50] Goldstein DE Little RR Lorenz RA Malone JI Nathan DM Peterson CM. Tests of glycemia in diabetes. Diabetes Care. (2004) 27(Suppl_1):S91–3. 10.2337/diacare.27.2007.s91 14693937

[51] Taboulet P Deconinck N Thurel A Haas L Manamani J Porcher R. Correlation between urine ketones (acetoacetate) and capillary blood ketones (3-beta-hydroxybutyrate) in hyperglycaemic patients. Diabetes Metab. (2007) 33:135–9. 10.1016/j.diabet.2006.11.006 17320448

[52] Stephens JM Sulway MJ Watkins PJ. Relationship of blood acetoacetate and 3-hydroxybutyrate in diabetes. Diabetes. (1971) 20:485–9. 10.2337/diab.20.7.485 4997333

[53] Jain SK Mcvie R Jaramillo JJ Chen Y. Hyperketonemia (acetoacetate) increases the oxidizability of LDL + VLDL in Type-I diabetic patients. Free Radic Biol Med. (1998) 24:175–81. 10.1016/s0891-5849(97)00213-x 9436628

[54] Jain SK Kannan K Lim G Matthews-Greer J Mcvie R Bocchini Jr JA. Elevated blood interleukin-6 levels in hyperketonemic type 1 diabetic patients and secretion by acetoacetate-treated cultured U937 monocytes. Diabetes Care. (2003) 26:2139–43. 10.2337/diacare.26.7.2139 12832326

[55] Rains JL Jain SK. Effect of hyperketonemia (Acetoacetate) on nuclear factor-kappaB and p38 mitogen-activated protein kinase activation mediated intercellular adhesion molecule 1 upregulation in endothelial cells. Metab Syndr Relat Disord. (2015) 13:71–7. 10.1089/met.2014.0101 25489974 PMC4398000

[56] Kanikarla-Marie P Jain SK. Hyperketonemia (acetoacetate) upregulates NADPH oxidase 4 and elevates oxidative stress, ICAM-1, and monocyte adhesivity in endothelial cells. Cell Physiol Biochem. (2015) 35:364–73. 10.1159/000369702 25591777 PMC4309197

[57] Kanikarla-Marie P Jain SK. 1,25(OH)2D3 inhibits oxidative stress and monocyte adhesion by mediating the upregulation of GCLC and GSH in endothelial cells treated with acetoacetate (ketosis). J Steroid Biochem Mol Biol. (2016) 159:94–101. 10.1016/j.jsbmb.2016.03.002 26949104 PMC4825694

